# Family function, coping style and well-being affect job burnout among telecommunication employees: a cross-sectional study in China

**DOI:** 10.3389/fpubh.2025.1567123

**Published:** 2025-05-21

**Authors:** Jingjing Song, Xinqing Xu, Hanzhong Zhang, Zhenyu Pan, Jinghua Zhu, Jiangang Shao, Liping Jia

**Affiliations:** ^1^Department of Psychology, Shandong Second Medical University, Weifang, China; ^2^Department of Network Center, Shandong Second Medical University, Weifang, China

**Keywords:** telecommunication employees, job burnout, family function, coping style, well-being, chain mediating effect

## Abstract

**Background:**

The rapid development of the digital economy has raised higher work demands for telecommunication employees, resulting in a growing prevalence of job burnout in this sector. Therefore, exploring the causes of job burnout among telecommunication employees and offering corresponding recommendations holds significant practical implications.

**Methods:**

Employing a stratified random sampling method, we surveyed 10,397 telecommunication employees from Shandong Province, China, achieving 8,018 valid responses (response rate: 77.1%). Using personal information forms and four scales. The collected data were processed and analyzed using SPSS 26.0.

**Results:**

Using partial correlation analysis, significant correlations (*ps* < 0.001) were found among family function, coping style, well-being, and job burnout. Coping style and well-being were identified as mediators between family function and job burnout, explaining 33.11% (indirect effect size = −0.101), and 24.59% (indirect effect size = −0.075) of the total effects, respectively. In addition, coping style and well-being play a chain mediating role between family function and job burnout, contributing 14.10% (indirect effect size = −0.043) to the total effects.

**Conclusion:**

This study highlights that family function not only directly impacts job burnout in telecommunication employees but also exerts an indirect influence through the chain mediating effect of coping style and well-being. Since this study employs a cross-sectional design, it can only reveal the correlations between these variables. Future research should focus on conducting longitudinal studies to further explore the causal relationships among the variables. The results of this study have important guiding significance for enterprises to pay attention to the family function of employees, cultivate positive coping style, improve their well-being, and reduce the level of job burnout of employees.

## 1 Introduction

With the rapid economic growth, individuals are facing increasing work and financial pressures, which are leading to greater physical and mental fatigue. This has resulted in a rising prevalence of job burnout (1, 2). Job burnout has been identified in various sectors, including healthcare, education, and law enforcement, where professionals report experiencing different levels of burnout (3–6). Job burnout occurs when there is an imbalance between the demands of work and the resources available to individuals (7). It is characterized by emotional exhaustion, a reduced sense of accomplishment, and other related behaviors, which can have negative consequences for both physical and mental health (8). In more severe cases, job burnout can lead to issues such as anxiety, depression, and sleep disturbances (9).

As the digital era rapidly progresses, the telecommunication industry plays a pivotal role in facilitating daily communication. However, with the industry’s increasing maturity and heightened market competition, job positions in the sector are becoming saturated, leading to a significant rise in employees’ workload and stress (10). Moreover, emerging technologies like artificial intelligence and cloud computing are prompting telecom companies to adopt more intelligent systems, creating the need for employees to acquire new skills, which in turn introduces additional challenges. Being a service-driven industry, telecommunication demands that employees maintain high levels of emotional engagement with customers to address their needs and resolve issues, which can result in emotional exhaustion. Furthermore, employees often face the monotony of performing repetitive tasks, leading to diminished job satisfaction and a heightened risk of burnout (11). Since service quality is critical in the telecommunication industry, the well-being and mental health of employees directly influence the efficiency and long-term success of the company. Consequently, identifying the main factors contributing to employees’ burnout and implementing strategies to improve job satisfaction and performance has become a vital concern for effective management in the sector.

In contemporary work environments, the relationship between employees’ professional and family lives has garnered significant attention in research within the fields of sociology and occupational health (12). The Job Demands-Resources (JD-R) model posits that all factors related to work can generally be categorized into two types: job demands and job resources (13). Job demands refer to factors in an individual’s work that require sustained physical and cognitive effort. Job resources, on the other hand, are material, social, and organizational factors that help achieve work goals, reduce the physical and psychological costs associated with job demands, or promote individual development (14). When the job resources that individuals obtain from the organization or society are insufficient to meet job demands over time, job burnout may occur (15). In this context, effective family function, as a social-level job resource (16), may play a crucial role in reducing individual job burnout. Specifically, family function, the way in which family members interact, cooperate, and address challenges, can profoundly impact individuals’ work experiences (17, 18). Family function refers to the capacity of a family as a social system to meet the needs of its members, resolving conflicts and providing support (19). Research has shown that individuals with strong family function are less likely to experience job burnout (20). A well-functioning family offers essential emotional and problem-solving support, which helps individuals cope with the stress they encounter in their professional lives (21). As the boundaries between work and personal life continue to become more fluid, the family has emerged as a key source of support for employees facing challenges in their careers. The quality of family function plays a crucial role in influencing employees’ mental well-being and work performance. Although it is evident that family function can serve as a predictor of job burnout, empirical studies examining this relationship among telecommunication employees are limited. The telecommunication sector is often characterized by a fast-paced work environment that requires employees to work overtime. A survey conducted in a telecommunication center found that 80.9% of telecommunication employees were obligated to work mandatory overtime (22). In such an environment, maintaining a balance between family and work commitments becomes especially difficult. Moreover, with the expanding regional scope of the telecommunication industry, employees’ frequent business trips further complicate the challenge. As such, family understanding and support become even more essential in managing the demands of a cross-regional work structure. Consequently, it is necessary to explore the role of family function in mitigating job burnout among telecommunication employees.

Coping style refers to the ways individuals manage stress, which can include proactive behaviors, such as problem-solving and employing effective strategies, or passive responses, such as avoidance, endurance, and withdrawal (23). Research has shown that external environmental factors significantly influence an individual’s coping style (24). Among these factors, family function is considered an important determinant (25). As a key support system, the family provides emotional support and a sense of security. A well-functioning family enables individuals to develop greater psychological resilience, helping them maintain a positive outlook when facing job-related stress (26). Additionally, an individual’s coping style plays a crucial role in the development of job burnout. Studies have found a significant relationship between coping style and job burnout, with negative coping identified as a risk factor for the occurrence of job burnout (27, 28). Telecommunication employees often deal with high levels of work pressure, which can negatively affect their family roles, such as spending less time with family or lacking emotional support, creating conflict between work and family. Employees who adopt proactive coping style and effectively manage the balance between work and family demands are more likely to reduce job burnout. Therefore, coping style may mediate the impact of family function on job burnout of telecommunication employees.

Family function plays a crucial role in an individual’s sense of well-being (29). When family function are disrupted, individuals may experience a loss of emotional support from their family, which can lead to feelings of loneliness and a decline in overall well-being (30, 31). Well-being is also a key factor in mitigating job-related burnout. Studies have shown that there is a negative relationship between well-being and job burnout, with higher levels of well-being linked to lower levels of burnout (32). Greater well-being not only improves job satisfaction but also plays a significant role in reducing emotional exhaustion and job burnout (33). The telecommunication industry often requires employees to work in shifts, including night shifts, which can make it challenging to balance work and family life. However, support and understanding from family members can not only enhance well-being in personal life but may also contribute to a reduction in job burnout. Thus, well-being may be another mediator in the relationship between family function and job burnout of telecommunication employees.

Relevant studies have found that coping style significantly impact an individual’s well-being (34). Individuals who adopt proactive problem-solving coping style tend to have higher psychological well-being, whereas those who resort to avoidant or accommodating coping style in the face of challenges experience lower levels of well-being (35). In workplace research, employees who are more inclined to positively cope with work stress exhibit higher levels of well-being (36). Accordingly, coping style and well-being may form a chain mediating mechanism through which family function affects job burnout of telecommunication employees.

In summary, although a considerable amount of research has been conducted on the relationships between family function, coping style, well-being, and job burnout, studies focusing on these variables within the telecommunication industry remain relatively scarce. Most studies either treat family function or coping style as independent variables, overlooking their interaction in the context of work stress, or fail to focus on specific industries, such as the telecommunications sector, in their research environment. Moreover, although research from the Job Demands-Resources perspective has effectively revealed the mechanisms underlying the formation of job burnout, most studies have primarily focused on external environmental factors within and outside the organization, i.e., the external resources of individuals, while giving less attention to the potential impact of an individual’s internal factors on job burnout (7). This study not only focuses on family function as an external resource but also examines internal coping style and well-being. It aims to provide theoretical guidance and intervention strategies for the telecommunications industry and similar sectors, helping employees improve family function, establish positive coping mechanisms, and enhance psychological well-being, thereby effectively reducing the occurrence of job burnout. To achieve this, we employed mediation analysis to assess the relationships among family function, coping style, well-being, and job burnout, proposing the following specific hypotheses:

*Hypothesis 1*: Family function can negatively predict job burnout of telecommunication employees.

*Hypothesis 2*: Coping style serves as a mediator between family function and job burnout among telecommunication employees.

*Hypothesis 3*: Well-being mediates the relationship between family function and job burnout among telecommunication employees.

*Hypothesis 4*: Coping style and well-being play a chain-mediated role in the relationship between family function and job burnout among telecommunication employees.

## 2 Methods

### 2.1 Study design, population, and sampling

The cross-sectional study was conducted from January to February 2024. We were commissioned by the Shandong Provincial Committee of the China Telecom Group Union to survey employees of Shandong Telecom. A stratified random sampling method was employed, with the total number of employees in each city determined based on the population proportion of each city in Shandong Province. Random sampling was then conducted within each city. A total of 10,397 telecommunication employees were selected to participate in the survey. Online questionnaires were distributed to the selected employees, with the telecommunication companies in each city of Shandong assisting in forwarding the surveys to ensure that all respondents were from the telecommunication industry. To minimize the impact of social desirability bias, we ensured that all respondents’ answers were anonymous and strictly maintained the confidentiality of the data, allowing them to freely express their genuine opinions. Additionally, we designed questions that were not directly related to the research objectives, reducing the likelihood of responses based on social expectations. Prior to completing the questionnaires, participants were informed about the study’s purpose and signed an informed consent form. A total of 8,018 valid questionnaires were successfully collected, yielding a response rate of 77.1%. The study was approved by the local medical ethics committee. The primary questionnaires used in this study included the Maslach Burnout Inventory (MBI), Family Assessment Device (FAD), Simplified Coping Style Questionnaire (SCSQ), World Health Organization Well-Being Index 5 (WHO-5), and demographic data (including gender, age, years of employment, labor relations, educational level, etc.).

### 2.2 Measures

#### 2.2.1 Job burnout

This study used the Simplified Chinese version of the Maslach Burnout Inventory (MBI) originally developed by Maslach and adapted by Li and Wu (33) to assess job burnout (37). The reliability and validity of this scale have been thoroughly validated in multiple studies with Chinese participants (38, 39). The scale includes three dimensions: emotional exhaustion, depersonalization, and reduced personal achievement. Higher scores indicate a higher level of burnout. The burnout scores are categorized as follows: emotional exhaustion score greater than 25, depersonalization score greater than 11, and reduced personal achievement score greater than 16. If any one of these thresholds is exceeded, it indicates mild burnout; if two thresholds are exceeded, it indicates moderate burnout; and if all three thresholds are exceeded, it indicates high burnout (40). In this study, the Cronbach’s *α* coefficient for the scale was 0.92.

#### 2.2.2 Family function

Family Assessment Device (FAD) compiled by Epstein et al. (19) and revised by Li et al. (41) was adopted to measure family function. The reliability and validity of this scale have been thoroughly validated in several studies involving Chinese participants (42, 43). The scale comprises seven dimensions: problem solving, communication, roles, affective responsiveness, affective involvement, behavior control, and overall function, totaling 60 items. It serves to assess fundamental family dynamics, including the closeness and communication capabilities among family members, and to identify potential family issues. Responses are rated on a 1–4 scale, where 1 indicates “very characteristic of my family” and 4 indicates “not characteristic of my family.” Scores range from 60 to 240, with higher scores indicating better family function after the data is converted. In this study, the Cronbach’s *α* coefficient for the overall scale was 0.93.

#### 2.2.3 Coping style

Coping styles were measured using the Chinese version of the Simple Coping Style Questionnaire (SCSQ) revised by Xie (44). The reliability and validity of this scale have been thoroughly validated in several studies involving Chinese participants (45, 46). It contains 20 items, which are divided into positive coping scale (1–12 items) and negative coping scale (13–20 items). Coping tendency score = positive coping standard score (Z) – negative coping standard score (Z). A score greater than 0 indicates that individuals tend to respond positively under stress. Less than 0, individuals tend to have negative coping style. In this study, the Cronbach’s *α* coefficient for the overall scale was 0.86.

#### 2.2.4 Well-being

Well-being was measured using the World Health Organization Well-Being Index 5(WHO-5) (47). The reliability and validity of this scale have been thoroughly validated in several studies involving Chinese participants (48, 49). This scale is widely used to assess individual mental and physical health. It consists 5 items, scored on a scale from 0 (not present) to 5 (constantly present), yielding a total score range of 0 to 25. Higher scores indicate better mental and physical health. In this study, the Cronbach’s *α* coefficient for the scale was 0.94.

### 2.3 Data analysis

Descriptive statistics and partial correlation analysis were conducted using SPSS 26.0 to examine family function, coping style, well-being, and job burnout. Subsequently, Hayes’ PROCESS macro Model 6 was employed to test the chain mediation effect of coping style and well-being in the relationship between family function and job burnout. All statistical tests were two-tailed, and the significance level was set at *α* = 0.05.

## 3 Results

### 3.1 Background information

The study sample (*N* = 8,018) comprised a larger proportion of males (52.3%) than females (47.7%). The incidence of mild burnout in males is 44.35%, moderate burnout is 6.44%, and high burnout is 1.77%. For females, the proportion of mild burnout is 49.35%, moderate burnout is 6.12%, and high burnout is 2.27%. The proportion of mild and high burnout is higher in females than in males, suggesting that female employees may face greater role conflict due to multiple responsibilities, which could lead to higher levels of burnout. The highest proportion of employees fell within the age bracket of 36–45 years (41.5%), followed by 29–35 years (28.7%), 28 years and younger (16.9%), and over 45 years (12.9%). Among employees with mild burnout, those aged ≤28 years have the highest burnout rate (50.66%). For moderate and high burnout, employees aged 29–35 years exhibit the highest burnout rates (7.70 and 2.57%, respectively). This age group may be experiencing pressure related to career development and higher professional demands. Regarding tenure, employees with 5 years or less (30.3%) constituted the largest group, followed by 11–15 years (23.2%), 6–10 years (19.0%), over 20 years (13.8%), and 16–20 years (13.7%). Among employees with mild burnout, those with 6–10 years of work experience have the highest burnout rate (51.25%). For moderate and high burnout, employees with 11–15 years of work experience exhibit the highest burnout rates (7.27 and 2.48%, respectively). Employees with ≥21 years of work experience show the lowest burnout rates, suggesting that extensive workplace experience may help reduce burnout. Outsourced employees accounted for a significantly higher proportion (72.5%) compared to those on contract (27.5%). The burnout rate among employees under the labor system is 40.06% for mild burnout, 5.44% for moderate burnout, and 1.18% for high burnout. For outsourced employees, the rates are 49.26% for mild burnout, 6.60% for moderate burnout, and 2.32% for high burnout. Outsourced employees experience significantly higher levels of burnout compared to those under the labor system, which may be attributed to factors such as job instability, lower compensation, and a lack of sense of belonging. The proportion of employees with bachelor’s degree (44.9%) is the largest, followed by an associate degree (36.8%), vocational diploma (8.3%), graduate degree or higher (5.7%), and high school diploma (4.3%). Among employees with mild burnout, those with a technical secondary school diploma exhibit the highest burnout rate (51.50%). For moderate and severe burnout, employees with a senior high school education exhibit the highest burnout rates (6.69 and 2.33%, respectively). Employees with a graduate degree or higher experience the least burnout, which may be associated with their higher professional status and better working conditions. The detection rates of mild, moderate and high burnout among telecommunication employees are shown in Table 1.

**Table 1:** Detection rates of mild, moderate, and high burnout among telecommunication employees (*N* = 8,018).

Variables	Type	Number of people	Mild burnout		Moderate burnout		High burnout	
			Number of people	Proportion (%)	Number of people	Proportion (%)	Number of people	Proportion (%)
Gender	Male	4,192	1859	44.35	270	6.44	74	1.77
	Female	3,826	1888	49.35	234	6.12	87	2.27
Age	≤28	1,356	687	50.66	90	6.64	28	2.06
	29–35	2,300	1,143	49.70	177	7.70	59	2.57
	36–45	3,329	1,500	45.06	183	5.50	66	1.98
	≥46	1,033	417	40.37	54	5.23	8	0.77
Years of employment	≤5	2,434	1,213	49.84	156	6.41	49	2.01
	6–10	1,526	782	51.25	104	6.82	37	2.42
	11–15	1858	869	46.77	135	7.27	46	2.48
	16–20	1,097	476	43.39	55	5.01	19	1.73
	≥21	1,103	407	36.90	54	4.90	10	0.91
Labor relations	Labor system employee	2,204	883	40.06	120	5.44	26	1.18
	Outsourced staff	5,814	2,864	49.26	384	6.60	135	2.32
Educational level	Technical secondary school	666	343	51.50	42	6.31	13	1.95
	Senior high school	344	169	49.13	23	6.69	8	2.33
	Junior college	2,954	1,517	51.35	196	6.64	64	2.17
	Undergraduate	3,598	1,550	43.08	221	6.14	74	2.06
	Postgraduate and above	456	168	36.84	22	4.82	2	0.44
Total		8,018	3,747	46.73	504	6.29	161	2.01

This table provides a detailed breakdown of the demographic statistics and the detection rates of mild, moderate, and high levels of burnout among 8,018 telecommunications employees.

### 3.2 Common method bias tests

An anonymous survey was conducted for the study, and Harman’s single-factor test was used to examine common method bias. The results indicated that in the unrotated exploratory factor analysis, 14 factors had eigenvalues greater than 1, with the highest factor explaining 21.92% of the variance (below 40%). Therefore, no significant common method bias was found in this study.

### 3.3 Correlation analysis of family function, coping style, well-being and job burnout among telecommunication employees

The results of the correlation analysis among family function, coping style, well-being, and job burnout in telecommunication employees are shown in Table 2. Controlling for demographic variables such as gender, age, years of employment, labor relations and educational level, significant correlations were observed between all pairs of variables.

**Table 2:** Correlation between family function, coping style, well-being and job burnout among telecommunication employees (*r* value)^a^.

Variables	Scale score (Mean ± SD)	1	2	3	4
1 Family function	172.83 ± 19.76	–			
2 Coping style	0 ± 1.33	0.556***	–		
3 Well-being	13.44 ± 6.79	0.467***	0.469***	–	
4 Job burnout	25.36 ± 13.68	−0.437***	−0.499***	−0.542***	–

^a^Partial correlation regression adjusted for study covariates including gender, age, years of employment, labor relations, educational level. ***p < 0.001.

### 3.4 Mediating roles of coping style and well-being in family function and job burnout

**Figure 1:**
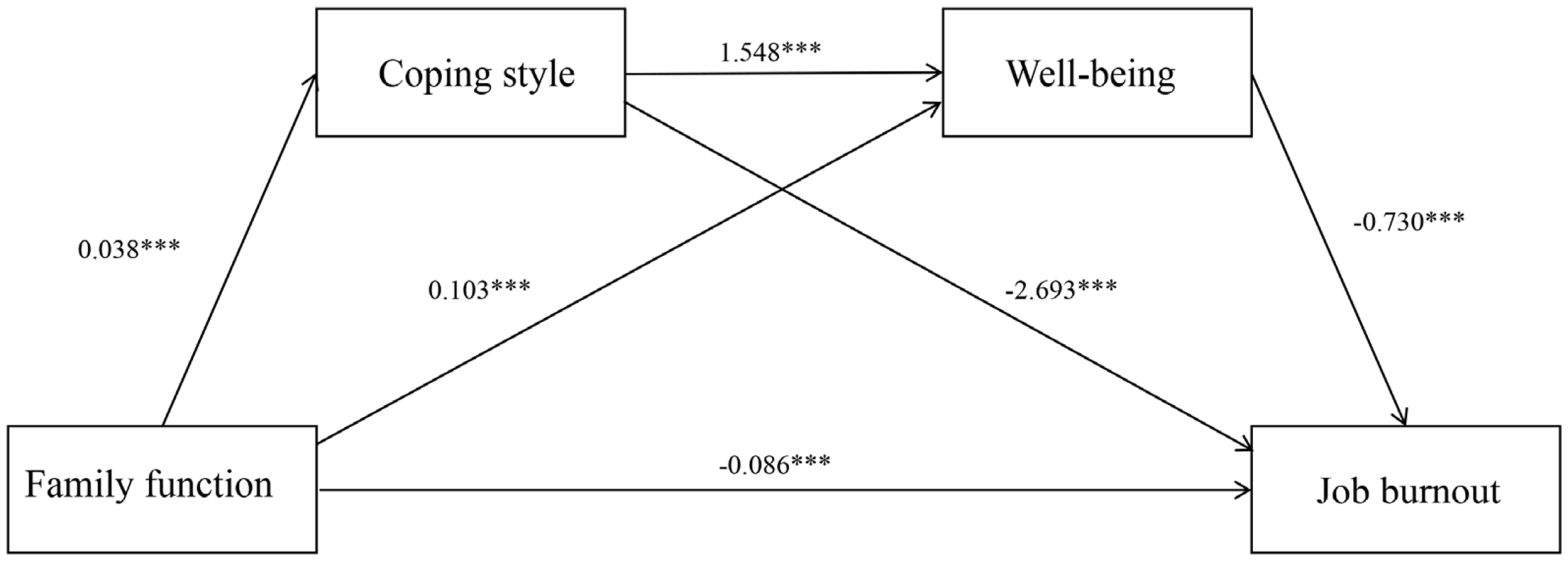
Diagram of the mediating effect model. Family functioning has a direct negative impact on job burnout (with a coefficient of −0.086) and influences job burnout through coping style and well-being. ***p < 0.001.

The correlation analysis results met the statistical criteria for further examination of mediation effects involving coping style and well-being. Bootstrap resampling with 5,000 iterations was employed to assess these effects using Hayes’ Model 6. A mediation effect was considered significant if the 95% confidence interval did not span zero. Family function served as the independent variable, job burnout as the dependent variable, coping style and well-being as mediator variables. Demographic variables including gender, age, years of employment, labor relations and educational level were controlled for in the analysis. Detailed path analysis outcomes are shown in Table 3 and Figure 1. The results indicate that family function has a direct negative impact on job burnout (with a coefficient of −0.086), and it exerts an indirect effect on job burnout through coping style (with coefficients of 0.038 and −2.693). Additionally, well-being has an indirect effect on job burnout (with a coefficient of 0.103 and −0.730). Furthermore, family function also has an indirect effect on job burnout through both coping style and well-being (with a coefficient of −0.219).

**Table 3:** Mediating effects of coping style and well-being of telecommunication employees in family function and job burnout.

Regression equation		Goodness of Fit index			Coefficient significance	
Dependent variable	Independent variable	*R*	*R* ^2^	*F*	*β*	*T*
Coping style	Family function	0.562	0.32	616.770***	0.038	59.852***
	Gender				0.010	0.385
	Age				0.155	7.430***
	Years of employment				−0.044	−3.110**
	Labor relations				−0.043	−1.393
	Educational level				−0.011	−0.800
Well-being	Family function	0.548	0.299	465.155***	0.103	26.230***
	Coping styles				1.548	26.595***
	Gender				0.300	2.292*
	Age				−0.052	−0.473
	Years of employment				−0.346	−4.736***
	Labor relations				−0.609	−3.820***
	Educational level				−0.425	−5.727***
Job burnout	Family function	0.629	0.384	623.05***	−0.086	−11.203***
	Coping styles				−2.693	−23.621***
	Well-being				−0.730	−34.815***
	Gender				1.265	5.153***
	Age				−0.159	−0.779
	Years of employment				−0.065	−0.474
	Labor relations				−0.171	0.573
	Educational level				0.419	3.001**

*p < 0.05, **p < 0.01, ***p < 0.001.

The results of the mediation analysis reveal significant indirect effects: coping style significantly mediate the relationship between family function and job burnout, explaining 33.11% of the total effect. Well-being also serves as a significant mediator between family function and job burnout, accounting for 24.59% of the total effect. Moreover, there is a notable chain mediating effect where both coping style and well-being jointly mediate this relationship, contributing to 14.10% of the total effect. Overall, the cumulative indirect effects represent 71.80% of the total effect, while the direct effect comprises 28.20%. Detailed findings are shown in Table 4.

**Table 4:** Analysis of indirect effects, direct effects and total effects.

Path	Effect	SE	Relative effect (%)	Boot LLCI	Boot ULCI
Family function → Coping style → Job burnout	−0.101	0.005	33.11%	−0.111	−0.092
Family function → Well-being → Job burnout	−0.075	0.004	24.59%	−0.083	−0.068
Family function → Coping style → Well-being → Job burnout	−0.043	0.002	14.10%	−0.047	−0.038
Indirect effect	−0.219	0.006	71.80%	−0.231	−0.208
Direct effect	−0.086	0.008	28.20%	−0.101	−0.071
Total effect	−0.305	0.007	–	−0.319	−0.292

Effect = Mediating effect value; SE = Standard error; Boot LLCI = Lower limit of bootstrap confidence interval; Boot ULCI=Upper limit of bootstrap confidence interval.

## 4 Discussion

This study measured job burnout among employees in the telecommunication industry in Shandong, China. The results indicated that 46.73% of telecommunication employees experienced mild job burnout, while 8.30% exhibited moderate to high burnout. These findings suggest that the job burnout situation among telecommunication employees in China is concerning. The results not only highlight the urgent need for telecommunication companies and industry managers to address employees’ mental health but also emphasize the importance of implementing effective intervention measures to reduce the negative impact of burnout symptoms on employees’ physical and mental well-being, as well as their job performance.

In this study, we found that outsourced employees and those with less work experience (≤10 years) exhibit significantly higher levels of job burnout. This phenomenon not only reflects the unique challenges faced by these groups but also provides strong data support for a deeper exploration of the mental health issues of different types of employees in the workplace. Outsourced employees experience significantly higher levels of burnout compared to labor system employees. Outsourced workers often face lower job security, limited opportunities for promotion, and greater uncertainty, all of which may contribute to increased work-related stress and psychological burden. Employees with less work experience (≤10 years) also report higher levels of burnout. Due to their lack of sufficient experience and psychological adjustment skills, they may be more susceptible to work-related stress. Therefore, businesses and managers should provide more career support for these employees. For outsourced workers, improving job security, enhancing benefits, and providing more career development opportunities are essential. For employees with less work experience, companies should assist them in managing stress through regular training, career planning, and psychological health support.

The examination of the relationship between family function and job burnout among telecommunication employees revealed that family function significantly negatively predicts job burnout, thus supporting Hypothesis 1. Specifically, when employees experience emotional support, warmth, and close relationships within their families, they are more likely to maintain positive emotions, which in turn reduces stress and burnout at work. This finding is consistent with existing research, which suggests that emotional support from the family and the overall health of family function can help employees alleviate workplace stress and improve job performance (50). In contrast, employees with poor family function may face greater emotional distress and role conflict in their family lives, which depletes their psychological resources and undermines their cognitive and emotional engagement at work (18). Although the negative effect of family function on job burnout (coefficient = −0.086) is statistically significant, the effect size is relatively small. We believe that, despite the small effect size, it still suggests that family function plays an important role in employees’ mental health. This small effect also reflects the potential impact of family function on employees’ emotions and burnout, particularly in the context of work-related stress. Good family support may provide employees with the necessary emotional resources, helping them better cope with the challenges at work. Therefore, our study emphasizes the crucial role of family function in mitigating job burnout. It suggests that, in addition to focusing on job performance, organizations should also pay attention to employees’ family environments, particularly the development of family support systems. Encouraging guidance through family relationships and providing training on work-life balance can help employees better cope with professional challenges. Organizations can offer employees professional family relationship counseling or coaching services to help improve communication skills among family members, enhance conflict resolution abilities, and strengthen emotional support.

The results of this study confirm Hypothesis 2, indicating that coping style mediate the relationship between family function and job burnout among telecommunication employees. Specifically, support and encouragement from family members can create a positive family atmosphere, which in turn improves individuals’ negative emotions and enhances their ability to cope with work-related stress, thereby effectively reducing job burnout. Previous research has shown that, when facing stress, a strong family support system can enhance an individual’s emotional regulation abilities and promote the adoption of positive coping style, such as seeking social support and problem-solving, rather than engaging in negative avoidance behaviors (51). However, employees with poor family function often lack this emotional support and, as a result, tend to adopt negative coping style when faced with challenges. Over time, these negative coping style can exacerbate the onset of job burnout (28). This phenomenon was further validated in our study, which also reinforced the mediating role of coping style between family function and job burnout. Although the positive effect of family function on coping style (coefficient = 0.038) is small, it is statistically significant. This relatively small effect size reflects the potential role of family support and emotional regulation in helping employees cope with stress. Even though the effect size is small in statistical terms, improving family function remains an effective approach to promoting employees’ mental health and reducing burnout. Organizations can regularly organize training sessions on stress management and emotional regulation to help employees understand how to use positive coping style, such as seeking social support, sharing stress with others, and problem-solving, rather than resorting to avoidance or negative coping mechanisms. This is particularly crucial in the telecommunications industry, where providing professional mental health support is essential due to the high-intensity nature of the work.

Furthermore, this study confirmed Hypothesis 3, which posits that subjective well-being mediates the relationship between family function and job burnout in telecommunications employees, individuals with stronger family intimacy typically report higher levels of subjective well-being. This finding is consistent with previous research, which has shown that individuals with closer family relationships report higher levels of well-being (52). Individuals with higher levels of well-being tend to exhibit greater emotional and psychological resilience, which leads to higher engagement and a more positive attitude at work, effectively reducing the incidence of job burnout (53). Individuals raised in dysfunctional family environments may develop negative cognitive frameworks, perceiving themselves as incapable of altering the difficulties in their work and personal lives. This sense of helplessness and disappointment exacerbates job burnout symptoms (54). This suggests that, in addition to strengthening family support systems, organizations can implement health and wellness programs, offering services such as health screenings, nutritional counseling, as well as stress management tools like meditation and mindfulness training, to help employees maintain both physical and mental well-being. Organizations can not only enhance employees’ sense of well-being and emotional regulation abilities but also assist them in achieving a better work-life balance, ultimately reducing the incidence of job burnout and improving overall employee well-being and job performance.

This study further reveals that coping style and well-being serve as a chain-mediated mechanism linking family function to job burnout among telecommunication employees, thereby confirming Hypothesis 4. A harmonious family environment is a critical aspect of life, as it boosts individuals’ confidence in problem-solving and enables them to address challenges with a more positive mindset (55, 56). Consequently, telecommunication employees with strong family function are more likely to adopt positive coping style in the face of work-related stress, which not only enhances their well-being but also helps mitigate work pressure, ultimately reducing burnout. In contrast, employees in environments with poor family function tend to rely on negative coping style when confronted with challenges. Their emotions are more vulnerable to negative influences, leading to increased feelings of anger, anxiety, or dissatisfaction. The accumulation of such negative emotions not only erodes individual well-being but also exacerbates the effects of work stress (57, 58). Consequently, family function influences job burnout by improving coping mechanisms and increasing well-being, thus providing a more comprehensive regulation of an individual’s psychological and emotional state. This helps individuals better manage work pressure and mitigate burnout. While the direct effect of family function on job burnout may be relatively straightforward and singular, its indirect effects operate through multiple psychological mechanisms, amplifying its impact. Although the chain mediation pathway (Family function → Coping style → Well-being → Job burnout) exhibits relatively small effect sizes, it shows statistical significance. Small effect sizes do not imply a lack of substantive meaning in the relationship; rather, they suggest that each path has a weaker effect individually, but their combined influence may have a cumulative impact on individual job burnout. The findings of this study have important practical implications for the intervention and prevention of job burnout. Specifically, understanding how family function, coping style, and well-being collectively influence job burnout can provide organizations with multidimensional psychological health intervention strategies, thereby helping to alleviate employee burnout and enhance overall well-being. In conclusion, organizations should consider regularly organizing activities that strengthen emotional bonds among family members.

Our research holds significant theoretical and practical implications. Theoretically, the study extends the understanding of the factors influencing job burnout, particularly within the framework of the Job Demands-Resources model. It further emphasizes the critical role of family function, as a form of social resource, in reducing job burnout. Additionally, this study expands the concept of individual internal resources by incorporating coping style and well-being, and proposes a mediating mechanism in which family function, coping style, and well-being contribute to job burnout. Practically, organizations should not only focus on employees’ work performance but also consider their family life, particularly the health of their family function. Organizations can assist employees in enhancing family function by implementing relevant policies, such as family relationship training and work-life balance guidance. This can improve employees’ emotional regulation and coping style, thereby reducing the risk of job burnout. Furthermore, organizations could introduce mental health initiatives, such as offering emotional support, meditation, and mindfulness training, to enhance employees’ well-being and help them better cope with workplace stress.

In conclusion, interventions aimed at reducing job burnout among telecommunication employees should employ a multifaceted approach. First, organizations should regularly organize training sessions and workshops focused on family communication and support to help employees improve communication skills among family members, encourage and support employees in fostering strong communication and support systems with their family members, ensuring that they receive emotional support when dealing with work-related stress. Secondly, organizations should provide training on how to identify and manage work-related stress effectively, teaching employees to use coping style such as deep breathing, meditation, and mindfulness to maintain a calm and composed attitude when faced with work pressure, thereby enabling them to handle stress appropriately. Furthermore, companies should prioritize enhancing employees’ overall well-being by fostering a supportive and understanding corporate culture, where employees can work in an environment free from stress and anxiety, thereby reducing burnout caused by a negative work environment. This atmosphere can be promoted through regular team-building activities, a culture of positive feedback, and social events, all of which contribute to improving employee enthusiasm and job satisfaction. Therefore, organizations should adopt a comprehensive approach that considers the role of family function, coping style, and well-being in reducing job burnout, providing employees with a more supportive, inclusive, and healthy work environment.

This is crucial for helping them manage the pressures and challenges in the workplace. Additionally, companies should prioritize enhancing employees’ overall well-being to boost their enthusiasm and job satisfaction. Therefore, organizations should adopt a holistic approach that takes into account the roles of family function, coping style, and well-being in reducing job burnout.

## 5 Limitations

Some limitations of this study should be considered while interpreting the findings. Firstly, this study is a cross-sectional study, which limits the ability to establish causal relationships and observe the long-term trends of job burnout among telecommunication employees. Since the data were collected at a single point in time, although the study found a negative correlation between family function and job burnout, we are unable to determine the causal relationship between these two variables—whether family function influences job burnout or whether job burnout affects family function. Future research should focus on conducting longitudinal studies to address these limitations. In addition, the study participants were employees specifically from local Province, which may restrict the applicability of findings to telecommunication professionals nationwide. To improve generalizability, future research could employ a stratified random sampling method across the country. Third, there may be unmeasured mediating mechanisms between the study variables, or individual differences among employees that could influence the research conclusions. Future studies should continue to explore variables closely related to employees.

## 6 Conclusion

In conclusion, family function not only has a direct impact on telecommunication employees’ job burnout but also exerts an indirect effect through coping style and well-being. Therefore, alleviating job burnout among telecommunication employees requires a joint effort between families and organizations. Family members should provide employees with sufficient support and trust, while organizations should consider employees’ work environment and mental health. Together, these efforts can enhance employees’ well-being, improve job satisfaction, and boost work efficiency.

## Data Availability

The raw data supporting the conclusions of this article will be made available by the authors, without undue reservation.
